# Visceral leishmaniasis–human immunodeficiency virus coinfection in a 52-year-old male in southwest Iran: a case report

**DOI:** 10.1186/s13256-023-04074-x

**Published:** 2023-08-09

**Authors:** Sasan Moogahi, Fateme Tadi Beni, Mehdi Tavalla, Maryam Fasihi-Karami, Forough Kazemi

**Affiliations:** 1 Infectious and Tropical Diseases Research Center, Health Research Institute, Ahvaz Jundishapur University of Medical Sciences, Ahvaz, Iran; 2 Student Research Committee, Ahvaz Jundishapur University of Medical Sciences, Ahvaz, Iran; 3 Department of Parasitology, Faculty of Medicine, Ahvaz Jundishapur University of Medical Sciences, Ahvaz, Iran

**Keywords:** Visceral leishmaniasis, Human immunodeficiency virus, Iran, *Leishmania*, Polymerase chain reaction, Case report

## Abstract

**Background:**

Leishmaniasis is a rare infectious disease observed in subtropical and tropical areas. This disease that demonstrates different clinical characteristics is caused by intracellular *Leishmania* protozoan. One of the important countries facing the incidence of this infectious disease is Iran. Recently, human immunodeficiency virus–*Leishmania* coinfection has been indicated in Iran.

**Case presentation:**

In the present case report, we show an atypical case of severe visceral leishmaniasis in a 52-year-old Iranian-Arab male with positive human immunodeficiency virus status. Leishmaniasis was detected by node biopsy and subsequently histopathology evaluations and confirmed by molecular methods.

**Conclusions:**

The current study was the first report of an atypical case of a patient with *Leishmania*–human immunodeficiency virus coinfection in southwestern Iran, which was not responsive to the treatment. Therefore, the health authorities should be aware of these reports, which require permanent clinical follow-up of the patients as well as effective treatments.

## Background

Leishmaniasis is a rare infectious disease observed in subtropical and tropical areas. This disease that demonstrates different clinical characteristics is caused by intracellular *Leishmania* protozoan [1]. One of the important countries facing the incidence of this infectious disease is Iran; almost all cities and/or areas of Iran have been faced with leishmaniasis [2]. This disease exists in several forms in Iran including visceral leishmaniasis (VL), cutaneous leishmaniasis (CL) and even post-kala-azar dermal leishmaniasis [3, 4]. Recently, human immunodeficiency virus (HIV)–*Leishmania* coinfection has been indicated in Iran [1]. This coinfection is an opportunistic disease in immunosuppressed people such as individuals with acquired immunodeficiency syndrome (AIDS) that shows specific characteristics, such as disseminated/diffused skin lesions and/or splenomegaly. In fact, immunosuppression in people with HIV is a major risk factor to alter clinical manifestations and even the treatment responses [5, 6]. Since the number of HIV and *Leishmania* infections has been increasing in Iran in recent decades and there is an overlap in both infections, the incidence of a coinfection will most likely increase [6, 7]. Hence, the current study was the first report of an atypical case of a patient with visceral *Leishmania*–HIV coinfection in southwestern Iran, which was not responsive to treatment.

## Case presentation

In May 2020, a 52-year-old Iranian-Arab male, a municipal employee, living in Dezful City, Khuzestan Province, southwest Iran referred to the Razi hospital affiliated to Ahvaz Jundishapur University of Medical Sciences for the evaluation of pain in abdomen. He was without any history of smoking, alcohol consumption, drugs, or underlying disease. Vital signs at the first visit were blood pressure 100/60 mmHg, pulse rate 70 beats per minute, respiratory rate 19 breaths per minute, and temperature 36.5 °C. On initial examination, pain in the epigastric region and left lower quadrant was reported. Moreover, splenomegaly (192 mm in size) was observed via ultrasound examination of the abdomen. The patient was admitted with suspicion of lymphoproliferative malignancy and bone marrow and examined by microscopic and flow cytometry methods; however, bone marrow was normal. In the next stage, the patient was tested for viral causes, which was negative for hepatitis B virus antigen (HBSAg) and hepatitis C virus antibody (HCV Ab), but he was positive for HIV. The patient underwent splenectomy, and then antiviral treatment was initiated with highly active antiretroviral therapy (dolatgravir + tenofovir + emtricitabine). Afterward, the patient was discharged from the hospital in good general condition.

**Fig. 1:**
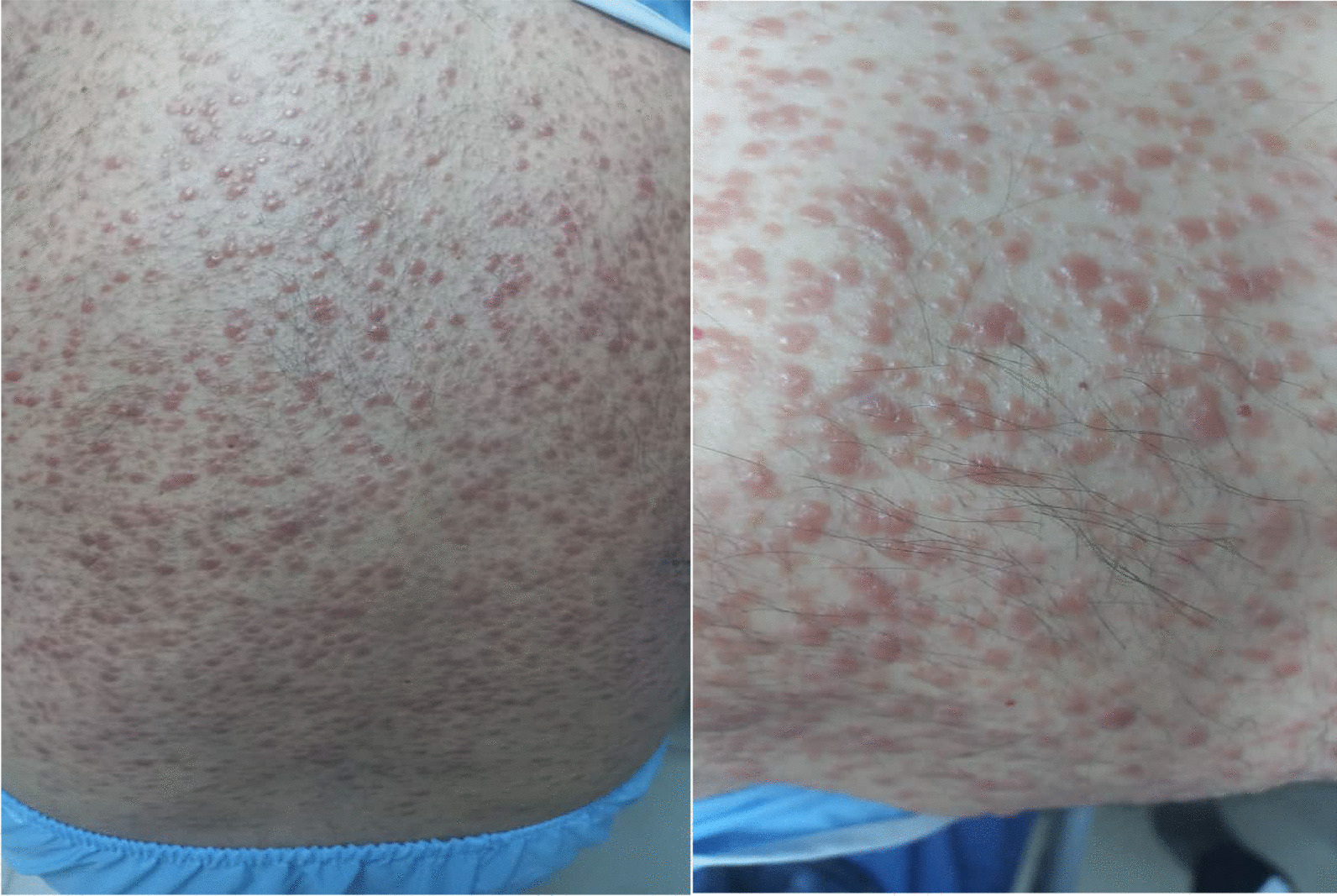
Clinical characteristics of the patient with human immunodeficiency virus–*Leishmania* coinfection on back

About 9 months later, he was referred to the Razi hospital again, because of large lymphadenopathies in the neck, axillary, and inguinal areas (after splenectomy). Then, the patient underwent a lymph node biopsy that indicated visceral leishmaniasis, and then was treated with intravenous amphotericin B at a dose of 5 mg per kilogram of body weight from day 1 to 5, then on days 10, 17, 21, 28, and 35. Due to not responding to the treatment, the patient underwent a lymph node biopsy again and was examined in terms of *Mycobacterium tuberculosis* (MTB), nontubercular mycobacteria (NTM), *Toxoplasma*, and *Leishmania*. Finally, visceral leishmaniasis was confirmed for the second time. The patient was again treated with amphotericin B (350 mg daily for 3 weeks, followed by weekly injections), and during the treatment, multiple, diffused, light-purple to reddish-brown nodular skin lesions were seen on the neck, chest, trunk, and abdomen. Clinical characteristics of the patient with HIV–*Leishmania* coinfection on the back are shown in Fig. 1.

**Fig. 2:**
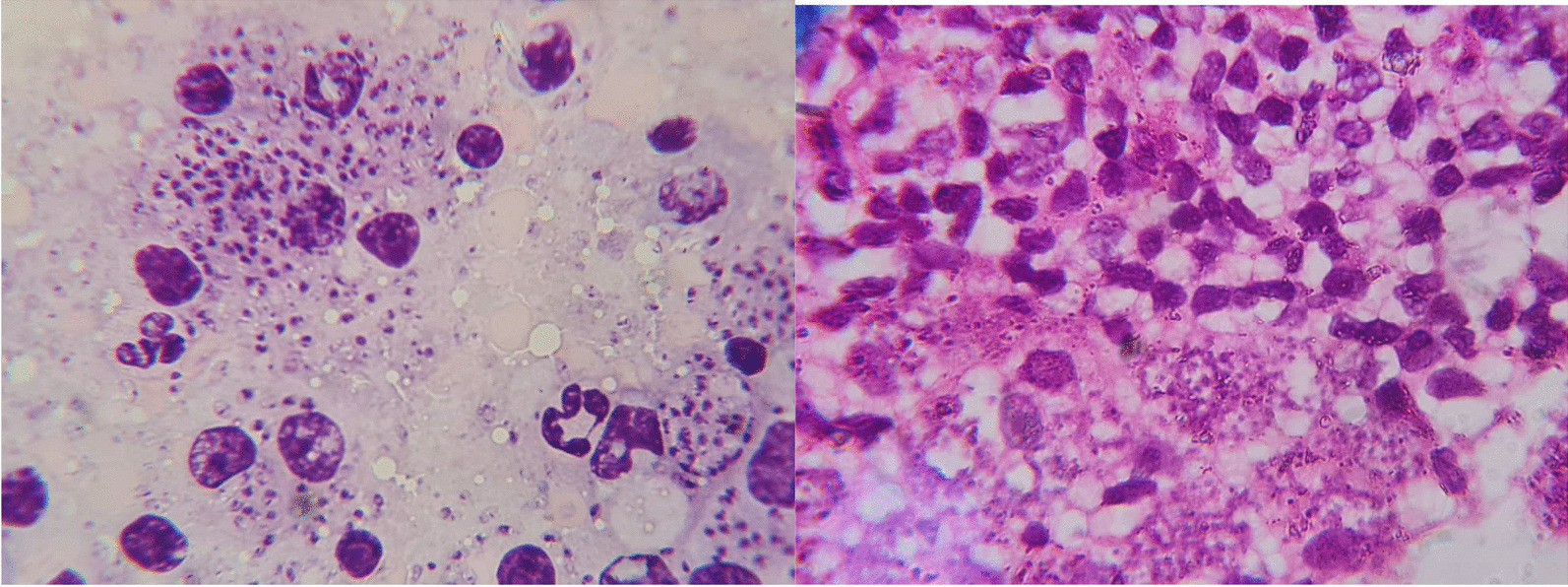
The arrow shows *Leishmania* bodies in the Giemsa-stained smear

**Fig. 3:**
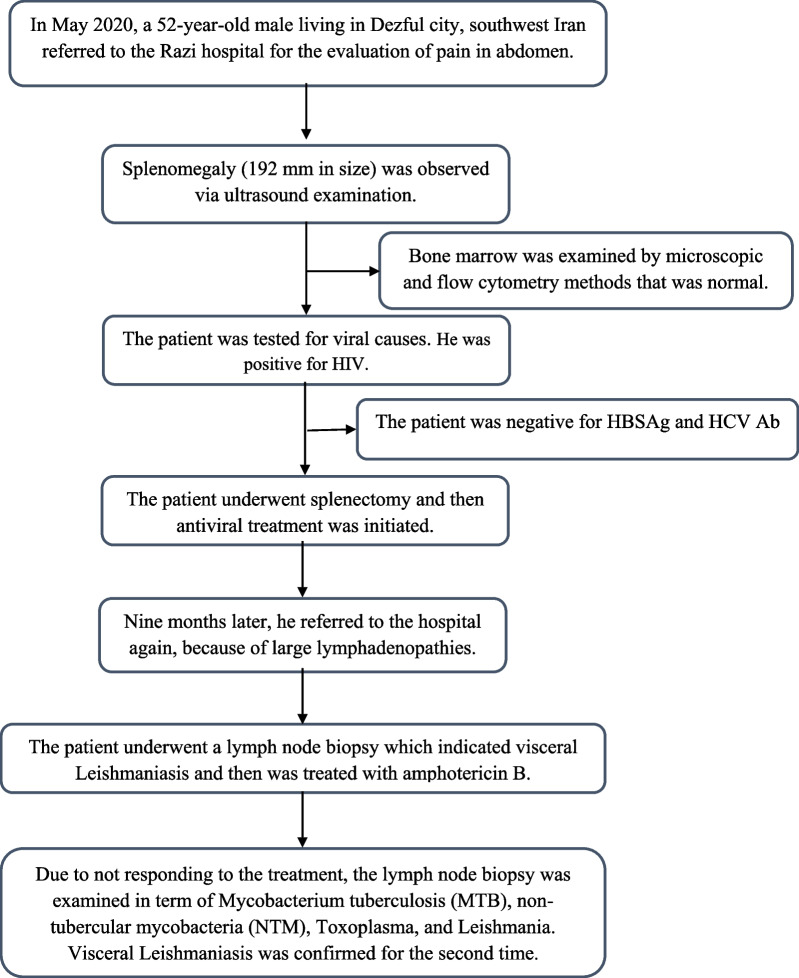
A summary of the clinical course of all follow-up

After 8 months, the patient was bedridden in the hospital. Vital signs at the first visit were as follows: blood pressure 110/60 mmHg, pulse rate 76 beats per minute, respiratory rate 21 breaths per minute, and temperature 36 °C. On neurological examination, the patient was alert and had no ptosis. Cranial nerve examination was normal. Submandibular and submental lymph nodes (38 × 13 and 34 × 12 mm in size, respectively) was observed via ultrasound examination. Furthermore, para-aortic, porta hepatis, and mesenteric lymph nodes (38 × 35 mm in size) were reported by computerized tomography (CT) scan. For evaluating the reasons, the laboratory tests were carried out with precision. Some of the significant items are presented in Table 1. Then, again, nodular skin lesions and lymph nodes underwent a biopsy and the specimens were positive and negative for leishmaniasis and acid-fast bacillus (AFB), respectively. At this time, the patient underwent the treatment with amphotericin B and the skin lesions were not removed yet. Due to the pansinusitis in the patient (observed in both clinically and imaging examination), the patient underwent a sinus biopsy for culture of fungi, bacteria, leishmaniasis, and typical and atypical mycobacteria. The results of polymerase chain reaction (PCR) and pathological methods showed that the sinus was negative for MTB and NTM; however, leishmaniasis bodies were detected in the sinus. Figure 2 shows *Leishmania* bodies in the Giemsa-stained smear. Finally, the patient was currently treating by Glucantime (20 mg/kg/day) and amphotericin (5 mg/kg/day), and unfortunately the skin lesions have been not removed yet. The patient was being followed up, but unfortunately he died. Figure 3 shows a summary of the clinical course of all follow-up. A written consent was taken from the patient for the publication of the present case study.

**Table 1:** Laboratory tests carried out to evaluate the patient with HIV–*Leishmania* coinfection

Tests	Results
WBC	900 cells per cubic millimeter
RBC	33,000 cells per cubic millimeter
Platelets	60,000 per microliter of blood
Hemoglobin	6.9 g per deciliter
AST	32
ALT	9
Alkp	148
Bilirubin total	0.5
BUN	8
Creatinine	1.5
ESR	100
Urinalysis	WBC: 7, RBC: 5, protein: 1+, pH: 7.1, hemoglobin: trace
Toxoplasma IgG	Negative
Toxoplasma IgM	Negative
CMV Ab	Negative
Wright	Negative
Coombs Wright	Negative
2ME	Negative
EBV IgG	Positive (19/68)
EBV IgM	Positive (12/18)

*WBC* white blood cells, *RBC* red blood cells,* AST* aspartate transaminase,* ALT* alanine transaminase,* Alkp* alkaline phosphatase,* BUN* blood urea nitrogen,* ESR* erythrocyte sedimentation rate,* CMV* cytomegalovirus,* 2ME* 2-Mercaptoethanol,* EBV* Epstein–Barr virus

## Discussion and conclusions

The current study was the first report of an atypical case of a patient with visceral *Leishmania*–HIV coinfection in southwestern Iran, which was not responsive to the treatment. In addition, the patient presented the cutaneous manifestations.It is notable that the clinical symptoms were consistent with cutaneous leishmaniasis as the patient had an intense immunocompromised condition.

AIDS/HIV occurs sporadically and is one of the main increasing disorders in Iran. Moreover, leishmaniasis (an endemic disease) is an opportunistic infection in people with AIDS [7]. The coinfection has been globally and increasingly seen in several countries including Brazil and East Africa and has even been detected in southern Europe [7, 8]. A zoonotic infection common is visceral leishmaniasis (VL or kala-azar), which is caused by *L. infantum* and *L. donovani* transmitted by the bite of hematophagous sand flies belonging to the genus *Lutzomyia* [9, 10]*.*

A limited number of the coinfections (HIV/leishmaniasis) has been reported in Iran, where the rare cases of HIV/cutaneous leishmaniasis were due to *L. major* and *L. tropica* [1, 11]. However, most of the cases were HIV/visceral leishmaniasis resulting from *L. infantum* [1, 6, 12]. It has been established that *L. infantum* (viscerotropic species) can diffuse to the skin; thereby this parasite may cause disseminated cutaneous leishmaniasis in HIV-positive patients. On the contrary, *L. tropica*/*L. major* (dermotropic species) can visceralize [12, 13]. Disseminated cutaneous leishmaniasis is defined via hematogenous dissemination of protozoans, leading to lesions with nodular, tumoral, and erythematous aspects as well as wide dermal disorders with the emergence of diffuse infiltration [14].

The clinical manifestations of leishmaniasis can mainly be atypical in HIV-positive patients, which includes disseminated cutaneous lesions, visceralization, intense side effects to drugs, and even resistance to medications as observed in the current case report [8]. One of the important mechanisms in this regard is a decrease in the levels of CD4^+^ T cell in HIV-positive patients [1, 2]. Moreover, both HIV and *Leishmania* separately impair T-cell function in patients [1, 8]. On the other hand, this parasite can stimulate the HIV genome transcription in the T cells through surface molecules such as lipophosphoglycan [1, 15]. For this reason, the protozoan acts as a cofactor in the pathogenesis of HIV and it has a tendency to disseminate to different regions of the HIV-positive patient’s body [1, 15]. Indeed, the cell-mediated responses (that is, lymphocytes of Th1 and Th2 CD_4_^+^) that are responsible for the clearance of *Leishmania* cannot be activated. This can lead to the reactivation of the latent parasite in HIV-positive patients, and leishmaniasis can increase the progression of AIDS through virus replication [1, 15].

One of the limitations of the present case report is that we did not determine the species of *Leishmania* in the HIV-positive patient using molecular techniques such as restriction fragment length polymorphism (RFLP). We have reported only one case of HIV–*Leishmania* coinfection in southwest Iran; however, it seems that the increasing coinfection has been widely underestimated. It is very notable that epidemiology of HIV–*Leishmania* coinfection has not been seriously understood, so further investigations are necessary in countries like Iran. As we know, *Leishmania* and HIV can result in the depletion of T-helper cells; accordingly, the coinfection promotes immunosuppression in patients. Hence, an effective and immediate treatment of both leishmaniasis and HIV infection is obligatory.

In conclusion, in some countries with high HIV and leishmaniasis-endemic conditions, especially in Iran, incidence of the coinfection along with atypical clinical symptoms is possible. Therefore, the health authorities should be aware of these, as they require permanent clinical follow-up of the patient as well as effective treatments.

## Data Availability

The datasets used and/or analyzed during the current study are available from the corresponding author on reasonable request.

## Abbreviations

HIV: Human immunodeficiency virus
CL: Cutaneous leishmaniasis
VL: Visceral leishmaniasis
AIDS: Acquired immunodeficiency syndrome
Th: T-helper
RFLP: Restriction fragment length polymorphism
AFB: Acid-fast bacillus
CT: Computerized tomography
MTB: *Mycobacterium tuberculosis*
NTM: Nontubercular mycobacteria
HCV Ab: Hepatitis C virus antibody
HBSAg: Hepatitis B virus antigen

## References

[1] Badirzadeh A, Mohebali M, Sabzevari S, Ghafoori M, Arzamani K, Seyyedin M (2018). Case report: First coinfection report of mixed *Leishmania infantum*/*Leishmania major* and human immunodeficiency virus–acquired immune deficiency syndrome: report of a case of disseminated cutaneous leishmaniasis in Iran. Am J Trop Med Hyg.

[2] Badirzadeh A, Mohebali M, Asadgol Z, Soong L, Zeinali M, Mokhayeri Y (2017). The burden of leishmaniasis in Iran, acquired from the global burden of disease during 1990–2010. Asian Pac J Trop Dis.

[3] Badirzadeh A, Mohebali M, Ghasemian M, Amini H, Zarei Z, Akhoundi B (2013). Cutaneous and post kala-azar dermal leishmaniasis caused by *Leishmania infantum* in endemic areas of visceral leishmaniasis, northwestern Iran 2002–2011: a case series. Pathog Glob Health.

[4] Mohebali M (2013). Visceral leishmaniasis in Iran: review of the epidemiological and clinical features. Iran J Parasitol.

[5] Parmentier L, Cusini A, Müller N, Zangger H, Hartley M-A, Desponds C (2016). Case Report: severe cutaneous leishmaniasis in a human immunodeficiency virus patient coinfected with *Leishmania braziliensis* and its endosymbiotic virus. Am J Trop Med Hyg.

[6] Pourahmad M, Hooshmand F, Rahiminejad M (2009). Cutaneous leishmaniasis associated with visceral leishmaniasis in a case of acquired immunodeficiency syndrome (AIDS). Int J Dermatol.

[7] Davarpanah M, Rassaei M. Presentation of AIDS with disseminated cutaneous and visceral leishmaniasis in Iran. Case Reports Infect Dis. 2015;2015. 10.1155/2015/563851 PMC4436502 26075117

[8] Calvopina M, Aguirre C, Cevallos W, Castillo A, Abbasi I, Warburg A (2017). Case report: coinfection of *Leishmania guyanensis* and human immunodeficiency virus-acquired immune deficiency syndrome: report of a case of disseminated cutaneous leishmaniasis in Ecuador. Am J Trop Med Hyg.

[9] Lindoso JA, Cota GF, da Cruz AM, Goto H, Maia-Elkhoury ANS, Romero GAS (2014). Visceral leishmaniasis and HIV coinfection in Latin America. PLoS Negl Trop Dis.

[10] Zijlstra EE. Precision medicine in control of visceral leishmaniasis caused by L. donovani. Front Cell Infect Microbiol. 2021:1007. 10.3389/fcimb.2021.707619 PMC8630745 34858865

[11] Jafari S, Hajiabdolbaghi M, Mohebali M, Hajjaran H, Hashemian H (2010). Disseminated leishmaniasis caused by *Leishmania tropica* in HIV-positive patients in the Islamic Republic of Iran. East Mediterr Health J.

[12] Shafiei R, Mohebali M, Akhoundi B, Galian MS, Kalantar F, Ashkan S (2014). Emergence of co-infection of visceral leishmaniasis in HIV-positive patients in northeast Iran: a preliminary study. Travel Med Infect Dis.

[13] Mohebali M, Malmasi A, Hajjaran H, Jamshidi S, Akhoundi B, Rezaei M (2011). Disseminated leishmaniasis caused by *Leishmania tropica* in a puppy from Karaj, Central Iran. Iran J Parasitol.

[14] Soares GHC, da Silva ABS, de Sousa Ferreira LS, Ithamar JS, de Alencar MG, Pereira SRF (2020). Case report: coinfection by *Leishmania amazonensis* and HIV in a Brazilian diffuse cutaneous leishmaniasis patient. Am J Trop Med Hyg.

[15] Calza L, D'Antuono A, Marinacci G, Manfredi R, Colangeli V, Passarini B (2004). Disseminated cutaneous leishmaniasis after visceral disease in a patient with AIDS. J Am Acad Dermatol.

